# Effect of Common Culinary Methods Practiced in Sri Lanka on the Nutrient Composition of Commonly Consumed Vegetables and Other Foods

**DOI:** 10.1155/2021/5537683

**Published:** 2021-07-10

**Authors:** H. G. N. Dewangani, B. M. Jayawardena, N. V. Chandrasekara, H. D. S. P. Wijayagunaratne

**Affiliations:** ^1^Department of Chemistry, Faculty of Science, University of Kelaniya, Sri Lanka; ^2^Department of Statistics & Computer Science, Faculty of Science, University of Kelaniya, Sri Lanka; ^3^Department of Physical Education, University of Kelaniya, Sri Lanka

## Abstract

In Sri Lankan traditional cooking, coconut and spices are incorporated to enhance the taste, flavor, and aroma. However, little attention has been given to assess the effect of these ingredients on the nutritional and chemical composition of the consumed food. The objective of this study was to ascertain the effect of traditional cooking methods on the chemical composition of vegetables, cereals and cereal-based foods, legumes, and selected nonvegetarian food varieties consumed in the daily diet. The results indicate that the addition of coconut milk (CM), coconut scraps, and coconut oil (CO) had a significant impact on the fat content of the prepared foods (*p* < 0.05). Cooking facilitated the incorporation of fat into food. According to the results, more percentage increases of fat content were observed in tempered string beans (97.51%) and cauliflower milk curry (96.6%). Data revealed that boiling helped to reduce the fat content in cereals and legumes. The cooked foods prepared using traditional recipes with CM, CO, or scraps have higher nutritional content than raw foods and have a significant nourishing potential that meets the daily energy requirements (*p* < 0.05). It can be concluded that the chemical composition of cooked food serves as a more realistic guideline in recommending dietary interventions in disease and weight management.

## 1. Introduction

In Asian countries such as Sri Lanka, the daily diet is mainly dominated by rice and vegetables. The combination allows adding variety to the everyday diet while providing nourishment and energy to sustain the quality of life. However, dietary patterns change over time. It is associated with many factors such as availability of food (seasons), income, food cost, preferences, cultural beliefs, and geographical and climatic changes [1].

Apart from rice and vegetables, locals also add meat products to their diets. Boiled legumes (lentils, cowpea, mung bean, and chickpea) and foods prepared with varieties of cereals (bread, roti, hoppers, noodles, string hoppers, and pittu) are other common supplements in Sri Lankan cuisine. The nutritional quality provided by any food will be determined by the method of cooking and processing. Nutrients can be lost by leaching or chemical degradation into the cooking matrix. Foods are mostly prepared based on their cost, convenience, safety, and sensory preferences [2]. A substantial amount of nutritional loss occurs during cooking. However, on the other hand, it improves food quality by deactivating microorganisms and antinutritional substances present in foods naturally or contaminant incorporated into food during the postharvest handling. Studies have shown that selecting the proper cooking method can enhance the availability of healthy nutrients [3]. Cooking increases digestibility by improving the food texture [4]. Cooking improves the sensory properties of foods such as taste, color, and aroma. Therefore, cooking has both beneficial and harmful effects. Coconut milk (CM), coconut scraps, and coconut oil (CO) are popular ingredients used in Sri Lankan cooking. Coconut serves as the major source of saturated fat in the diet [5]. Lauric acid is a major fatty acid present in coconut fat. Researchers have found that monolaurin, which is chemically derived from laurin, has antiviral and antibacterial effects on the human body [6]. CM has been reported to have many medicinal properties, including antioxidant, cardioprotective, anticholecystitic, anticancer, antithrombotic, antidermatophytic, immunostimulatory, antifungal, and antidiabetic effects [7]. In addition, CM is rich in potassium, sodium, calcium, magnesium, phosphorus, iron, and selenium. Thus, CM helps to reduce anemia and joint inflammation and promote the efficacy of the prostate gland. The use of CM has been proven beneficial in lowering LDL cholesterol levels [8]. Hence, the use of CM in cooking can lead to various changes in the nutrient content and the organoleptic properties of food.

Vegetables play a pivotal role in the human diet because of their nutritive value [9]. Vegetables are an excellent source of essential macronutrients, micronutrients, and dietary fiber. Generally, vegetables contain a small number of calories from fat [2]. Some vegetables can be eaten in raw form, but usually, many vegetables are cooked in different ways before consumption, such as boiling, roasting, frying, and grilling. In Sri Lanka, popular culinary practices are to prepare vegetable curries using CM. CM enhances the flavor of vegetables and improves palatability and acceptability.

The consumption of foods made from wheat flour and rice flour has become a popular choice as the main meal instead of rice in urban areas. Apart from bread other cereal-based foods are prepared based on traditional recipes. Coconut flatbread (roti), local rice flour spaghetti (string hoppers), steamed rice flour with coconut (pittu), and local pancakes (hoppers) are typical varieties of food consumed in the daily diets of locals. Most of these items are prepared from processed wheat flour or rice floor; hence, these are classified as high-glycemic index (GI) foods. Approximately 65% of locals consume starch servings well above the recommended level [10, 11]. In order to improve the nutritional quality of the food, supplementing wheat flour with millet flour (*Eleusine coracana*) or soy flour is recommended in order to reduce the GI [12–14]. Consumption of boiled legumes (chickpea, cowpea, and mung beans) for breakfast is another popular breakfast option adopted by locals. These are categorized as low GI foods [15]. Legumes are a good source of carbohydrates, dietary fiber, and protein, and locals consider these as a healthy breakfast option compared to cereal-based food [12]. Proximate nutrient analysis of foods plays a significant role in the assessment of nutritional quality. Since there are considerable variations in culinary practices globally, studies on consumable food are quite extensive [16]. In Sri Lankan culinary practices, condiments such as cinnamon, ginger, cloves, spice mixtures, cardamoms, coriander, mustard, pepper, turmeric, and curry leaves are added to improve the taste, flavor, and nutritional status. There is little or no information available on the effect of various cooking methods on the nutritive value of food consumed in Sri Lanka. Sri Lankans use a considerable amount of CM, CO, and coconut scraps in culinary practices. The nutritional impact of adding coconut-based products during food preparation is important because there is a high prevalence of cardiovascular diseases and diabetes in Sri Lanka. Hence, the present study is aimed at determining the effect of adding coconut-based products during cooking on the proximate and nutrient contents in traditional Sri Lankan diets.

## 2. Methodology

### 2.1. Sample Selection

Forty-three food items, including different vegetables, green leaves, legumes, meat, eggs, and fish products, and common flour-based products consumed for breakfast in a typical daily diet of Sri Lankans were included in this study. The details of each plant-based vegetable with the scientific names, vernacular names, and edible parts of the plant are listed in Table 1. Most of the samples were analyzed in both raw and cooked forms.

### 2.2. Cooking Treatments

Local recipes, commonly practiced using CM, CO, and scraped coconut, were used to prepare the food items. The food preparation methods were standardized to ensure that the composition of foods prepared on different days did not vary. In brief, milk curry vegetables (250 g) were prepared with the addition of 400-600 mL of CM. In the preparation of tempered vegetable curries, 15-20 mL of CO was used. All vegetables, including green leaves and nonvegetable samples, were cooked until the required palatability and consistency were achieved.

A 250 g sample of each food legume (chickpea, cowpea, and mung bean) was soaked in 1.5 L of water for 12 h and followed by draining the excess water. The legumes were washed thoroughly, and 800 mL of water was added and cooked on a stove for 60-90 min until the same degree of tenderness of each legume was achieved. To prepare coconut roti, wheat flour (250 g), required amounts of salt (3.5 g), water, and scraped coconut (200 g) were mixed well and kneaded slightly into a ball. The dough was then divided into equal size portions. Each dough portion was flattened on a lightly oiled surface in the shape of circles. Roti was placed in a pan and heated on a mild flame for 4-5 min. Roti was turned upside down in the pan when the color turned brown, and the other side was cooked for another 4-5 min. Dried noodles (250 g) were broken into small pieces (3-5 cm in length) and boiled in 800 mL of water for 10 min with sporadic stirring, and salt was added to achieve the required taste. The excess water was drained. Sweet potatoes (250 g) were cut into small pieces (3 cm). Water (800 mL) was added to submerge the pieces and boiled. The remaining water was discarded once a soft consistency was achieved. Coconut sambol (coconut kernel salad) was prepared by adding freshly shredded coconut (250 g), chili powder (20 g), salt (5 g), 4-5 raw garlic cloves, one small red onion (20 g), and lime (5 mL). All the ingredients were mixed thoroughly to make a paste with the appropriate texture. Bread samples were collected from local bakeries. In preparing the samples, cooking time and temperature were maintained until the required soft consistency and palatability were achieved. All the ingredients were purchased from local supermarkets. Even though standardized methods were adopted to prepare the food, in certain instances, the same food prepared under the same condition was different in physical appearance including color. The reason for the observed difference could be due to the different maturity levels and geographical variation of the raw food used for cooking.

### 2.3. Collection and Preparation of Raw Foods

Commonly consumed raw, fresh vegetables and other food items were purchased from local supermarkets from different regions in Sri Lanka. Samples were cleaned and washed with running tap water and distilled water to remove any impurities present and blotted dry to remove surface moisture. The air-dried samples were cut into small pieces and oven-dried in glass dishes at 55°C for 8-24 h to a constant weight.

Cooked food samples and raw food samples were oven-dried at 55°C for the same duration. The samples were then ground to a fine powder and stored at -20°C in separate, air-tight plastic containers until analysis.

### 2.4. Proximate Analysis of the Samples

Proximate chemical analysis (moisture, crude protein, fiber, ash, and crude fats) was carried out on both raw and cooked forms of dried samples following the official methods described by the Association of Official Analytical Chemists with minor modifications [17]. Standard analytical protocols followed by most food analysis studies were used in this study to make the results comparable with previous results. The moisture and ash contents were determined using the weight-difference method. The nitrogen values were determined by the Kjeldahl method, involving digestion, distillation, and finally titration of the sample, and it was converted to the protein by multiplying by a conversion factor relevant to each food (vegetables: 4.4, muscle foods: 5.6, cereals, legumes, and wheat-based foods: 5.4) [18]. Fat content was determined by the soxhlet extraction method, and the formic acid method was used to determine the fiber content. Finally, the carbohydrate content was determined using the difference method. The gross energy content of the samples was computed by multiplying the percentage values of carbohydrates, lipids, and proteins by a relevant factor for each proximate constituent.

#### 2.4.1. Moisture Content

The AOAC oven drying method was used for moisture analysis. Approximately 5.0 g of sample was weighed, placed in an aluminum container, and dried for 3 h at 105°C in an air-drying oven. The samples were then transferred to a desiccator to cool to constant weight:

(1) $$\begin{array}{c} \text{Percent moisture }\left(\%M\right)=\frac{\left(\text{wet weight}-\text{dry weight}\right)}{\text{wet weight}}\times 100. \end{array}$$

#### 2.4.2. Ash Content

Approximately 2.0 g of sample was placed into a dry, preweighed crucible dish that had previously been weighed and then inserted into a muffle furnace (digital muffle furnace, DMF-03, Human Lab. Inc., Korea) and incinerated at 550°C for 5-6 h. The sample was then transferred to a desiccator to cool to room temperature and then weighed:

(2) $$\begin{array}{c} \text{Percentage}\%\text{ash}=\frac{\text{ash weight}}{\text{wet weight}}\times 100. \end{array}$$

#### 2.4.3. Protein Content

A 2.0 g sample was digested with conc. H_2_SO_4_ (98% *v*/*v*, 20 mL) and the catalyst mixture (1.1 g) in a digestion flask (Gerhardt) for 3-4 h until a clear greenish solution appeared. The digested sample was then allowed to cool. Distilled water (20.0 mL) was then added to the digest. The sample was transferred to the auto Kjeldahl system (Gerhardt Vapodest 30 Kjeldahl distillation system). The mixture was distilled with the automatic addition of NaOH (45%), and the liberated ammonia was collected in a volumetric flask containing 20 mL of 2% boric acid mixed with the indicator. This was titrated against 0.01 M HCl solution. The blank was titrated in the same way but without the sample. The amount of crude protein was calculated by multiplying the percentage of nitrogen in the digest by the relevant conversion factor for each food:

(3) $$\begin{array}{c} N\%=\frac{\left(V_{\text{s}}-V_{\text{b}}\right)\times N_{\text{HCl}}\times 14.01\times 100}{W\times 100}, \end{array}$$

where *V*_s_ is the sample titer, *V*_b_ is the blank titer, *N*_HCl_ is the molarity of HCl, and *W* is the sample weight:

(4) $$\begin{array}{c} \text{Protein}\%=N\%\times \text{conversion factor}. \end{array}$$

#### 2.4.4. Fat Content

A 5.0 g sample of the dried, powdered sample was placed in an extraction thimble made of filter paper (Whatman No. 1). The sample was ether extracted using a soxhlet apparatus for 5-8 h. The fat extract was collected into a clean, dry preweighed flask. Then, the ether was evaporated from the extract, and the remaining residue was weighed. The weight of fat was determined by the difference between the final and initial weights of the flask. The crude fat content was estimated as g/100 g of the dry weight of the sample:

(5) $$\begin{array}{c} \text{Crude fat}\%=\frac{W_{2-}W_{1}}{W_{s}}\times 100, \end{array}$$

where *W*_1_ is the weight of the empty flask, *W*_2_ is the weight of the flask with fat content, and *W*_s_ is the weight of the sample.

#### 2.4.5. Fiber Content

The fiber content was determined using the formic acid method. A 2.0 g sample was weighed into a boiling tube containing 20 mL of formic acid (80%, *v*/*v*). The vial containing the sample was placed in a boiling water bath for 75 min. The vial was allowed to cool, the digest was filtered, and the resulting residue was transferred into a crucible. The crucible containing the sample was dried at 105°C for 1 h and weighed (*W*_1_). The residue was incinerated at 450°C for 30 min, cooled in a desiccator, and weighed (*W*_2_). The crude fiber content was determined by calculating the difference between the two weights.

#### 2.4.6. Carbohydrate Content

The sum of the percentage values of moisture, ash, crude protein, and crude fat was subtracted from 100 to determine the carbohydrate content [9]:

(6) $$\begin{array}{c} \text{Carbohydrates}\%=100-\left(\text{moisture}\%+\text{ash}\%+\text{protein}\%+\text{fat}\%\right). \end{array}$$

#### 2.4.7. Total Energy

The total energy/caloric values of the food samples were calculated by multiplying the percentage values of carbohydrates, lipids, and proteins by a factor of 4, 9, and 4, respectively [19]. Energy values were calculated and reported in the kcal unit.

(7) $$\begin{array}{c} \text{Energy }\left(\text{kcal}\right)=4\times \left(\text{g protein}+\text{g carbohydrate}\right)+9\times \left(\text{g lipid}\right). \end{array}$$

### 2.5. Data Analysis

All analyses were carried out in three replicates to evaluate the nutritional value of food commonly consumed by locals in Sri Lanka. Twenty-three fresh vegetables and their cooked forms, four green leafy vegetables, four different kinds of cereals and legumes, seven nonvegetarian food items, three different breakfast foods, and other commonly consumed foods were analyzed with a total of forty-three samples. Statistical analysis was performed using the Statistical Package for the Social Sciences (SPSS) software for Windows version 22.0 (IBM SPSS Inc., Chicago, Illinois, USA). Data from the experiments are presented as the mean ± standard deviation. Significant differences in nutrition values between raw and cooked foods were tested using a *t*-test and one-way ANOVA followed by Tukey's multiple comparison post hoc test. Before the analysis, the normality of data was assessed using the Shapiro-Wilk test. In all statistical analyses, a 5% level of significance was considered.

## 3. Results and Discussion

The proximate analysis results showed variation in the composition of biopolymers (fiber, protein, and carbohydrate) and other contents (fat, ash, and moisture). The macronutrient composition of the selected, widely consumed vegetables is shown in Table 2.

### 3.1. Vegetables

Significant changes in the proximate constituents were observed after cooking. The values of moisture content in raw vegetables ranged from 95.79 g to 68.70 g per 100 g of dry weight (DW). The high moisture content of vegetables facilitates the digestion process, but on the other hand, high moisture content reduces the shelf life of vegetables by enabling microbial activities [20]. Vegetables are more often consumed in cooked forms, although some are used in salad preparation. The results indicated that cooking using CM slightly increased the moisture content of some vegetables (carrot, drumstick, lotus root, potato, and ash plantain). This may be due to the absorption of water during cooking. The results indicate that tempering can cause a remarkable reduction in the moisture content of some vegetables.

Protein content of the raw vegetables was very similar and very low. Raw mushroom (3.24 g per 100 g of DW) and cauliflower (3.01 g per 100 g of DW) showed the highest protein content compared to the other vegetables. The *t*-test indicated significant differences (*p* < 0.001) between the crude protein content of raw and cooked vegetables. Cooking causes a substantial loss of protein. The decrease in protein content could be due to the high temperatures used during cooking which may cause protein denaturation and leaching. Some vegetables' (beetroot, snake gourd, and okra) protein content increased after cooking, although this increase was not significant.

The mineral content of a food is represented by its ash content [19]. The amount of minerals present in raw vegetables ranged from 410 mg in lufa to 1740 mg per 100 g of DW in string beans. The changes observed in the mineral content of cooked and raw vegetables did not follow a unique pattern. But the values obtained were comparable to the data reported in several studies [9, 19, 21]. In general, minerals of plant origin are less bioavailable than minerals of animal origin [19]. However, minerals from plant sources continue to play an essential role in maintaining normal body functions. In addition to that, in Sri Lankan culinary practices, locals add condiments while cooking, such as onion, garlic, curry leaves, fenugreek, chili, curry powder, mustered, and turmeric. These condiments may add a substantial amount of minerals and vitamins to the daily diet.

Vegetables are a good source of fiber. The fiber content was significantly different in cooked and raw vegetables. Some vegetables were found to be rich in fiber. Raw lassia had the highest fiber content (22.07 g per 100 g of DW) compared to the other vegetables analyzed. Raw lotus root (4.83 g per 100 g of DW), raw string bean (5.03 g per 100 g of DW), and raw banana blossom (6.13 g per 100 g of DW) can also be identified as rich sources of fiber. Except for pumpkin, beetroot, green beans, and knolkhol, all other vegetables showed a significant (*p* < 0.001) increase in dietary fiber values when cooked. It is documented that dietary fiber intake could reduce body cholesterol levels and improve bowel movement [9]. Hence, the addition of lassia, lotus root, banana blossom, and string bean to the diet would provide the required dietary fiber content to maintain human health and help in cleansing the digestive tract.

Plant fibers can be divided based on solubility, viscosity, and fermentability. Soluble fiber (dextrin and oligosaccharides) generally dissolves in water, while insoluble fiber (hemicellulose, cellulose, and lignin) does not dissolve in water. According to most previous studies, insoluble fiber content is not altered by cooking, and in some instances, a slight decrease has been observed in the insoluble fraction [22]. The observed changes in fiber content were due to the breaking down of the large polymers into more soluble forms during prolonged exposure to heat [23].

**Figure 1:**
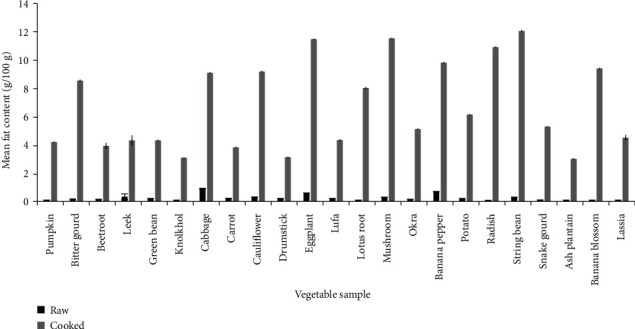
Changes in fat content in different varieties of raw vegetables and cooked vegetables. Results represent the mean ± SD (*n* = 6) at a 5% level of significance.

Raw vegetables are a poor source of fat. As illustrated in Figure 1, all vegetables showed a significant increase in fat content after cooking (*p* < 0.001). Tempered vegetables showed higher content of fat than vegetables prepared using CM. The fat contents of tempered eggplant and radish were not significantly different (*p* < 0.05). This significant increase in fat content can be attributed to the oil being absorbed into the vegetables after water loss from the tissues due to evaporation [14]. There was a significant variation in the fat content (*p* < 0.001) in all vegetables. However, there were no significant differences (*p* < 0.05) in the fat content of lassia (curry), lufa (curry), green bean (curry), knolkhol, drumstick, and ash plantain's milk curries. The physical nature of the food might play an essential role in the fat-absorbing process. Each vegetable may have its own physical and biochemical characteristics, which contribute to fat absorption. The results are comparable to previous observations reported by Silva et al. [14], where an increase in the fat content was reported in foods cooked with CM.

CM can be identified as a primary source of culinary fat in the Sri Lankan diet. According to the literature, chemical composition of CM is moisture 50.0 g-54.1 g, fat 39.2 g-40 g, protein 2.8 g-4.4 g, ash 1.0 g-1.5 g, and carbohydrate 5.5 g-8.3 g [24]. It was observed that the addition of CM increased the fat content by an average of fivefold when compared to the fat content in raw vegetables while CO increased the fat content by an average of ninefold compared to the raw form. In the case of curry, CM and heat treatment increased the *in vitro* availability of essential nutrients. It has been reported that vitamin A content is increased when vegetables are prepared with the addition of CM [25]. Hence, adding CM and preparing curries seem to be the best method to adopt in preparing vegetables.

The consumption of dietary fat in any form is linked with the incidence of coronary artery disease. A study on dietary intake of CM and the risk of coronary heart disease has shown that natural coconut fat in the diet does not cause any detrimental effects on the lipid profile [8]. However, a high intake of muscle foods (protein), dietary cholesterol, and less amount of plant-derived carbohydrates has been identified as a risk factor for coronary heart disease [26]. In considering our results and what is reported in the literature, it can be concluded that the use of a moderate amount of CM as a cooking medium may provide many health benefits. The proximate composition of food can be affected by many factors, including vegetable species, climatic conditions, and nature of the soil, application of natural or artificial manure/fertilizer to the plant growing soil, and postharvest treatments [27].

### 3.2. Green Leaves

**Figure 2:**
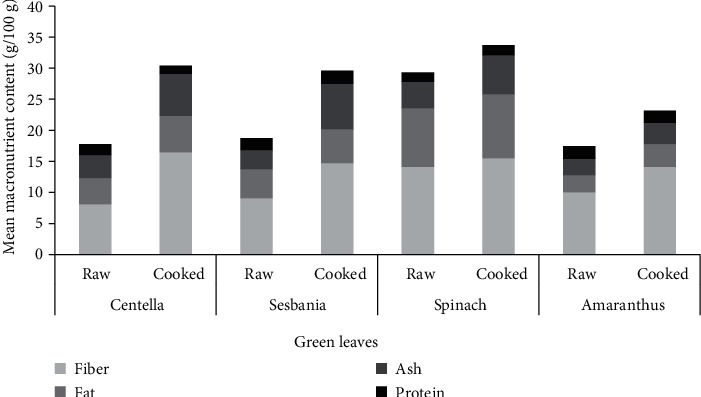
Illustration of changes of the proximate constituent in raw and cooked green leaves.

The proximate nutrition content of four commonly consumed green leafy vegetables is presented in Table 3. The statistical analysis result showed significant change (*p* < 0.001) in nutrition content of raw and cooked green leaves. Comparison of proximate values of raw and cooked green leaves is illustrated in Figure 2. The values of moisture content in raw green leaves ranged from 67.78 g to 81.80 g per 100 g of DW. These results are comparable to the moisture content values reported in commonly consumed green leaves in Sri Lanka [14].

The ash content, which is a measure of the level of inorganic elements present within the sample, showed values ranging from 2660 mg to 4340 mg per 100 g of DW. There is a significant increase in the ash content after cooking. The increase of the ash content can be attributed to the addition of condiments into green leafy vegetables in cooking. Locals adopt low heat in cooking green leaves and try to preserve the green color and the vitamins present in the leaves. There is a considerable amount of crude fiber in all four types of green leaves studied; hence, green leaves are a rich source of dietary fiber. Adding green leaves to the daily diet improves gastrointestinal health.

Fresh green leaves usually contain a certain amount of fat. Centella leaves are used to extract oil for ayurveda purposes [28]. In comparing the fat content between raw and cooked samples, cooked samples had greater content of fat. The scrapped coconut added in the preparation of green leaves salads increased their fat content [14]. The fat contents reported are similar to the previously reported values [29].

### 3.3. Cereals and Legumes

Cereals and legumes are good sources of protein, fiber, and carbohydrates. The results of the proximate composition of raw and cooked cereals and legumes are given in Table 4. Comparisons of raw and cooked legumes and cereals indicated a significant difference in their proximate compositions at *p* ≤ 0.001.

The amount of moisture in raw legumes varied from 12.58 g in cowpea to 89.66 g per 100 g of DW in dal. The fat content in all legumes was low, and the values lie in the range of 0.16 g and 6.27 g per 100 g of DW. Protein content in raw legumes ranged from 3.16 g to 22.19 g per 100 g of DW. Cooked legumes showed a significant reduction in crude protein than raw legumes. Among the legumes analyzed, cooked dal and chickpea contain a significantly high (*p* < 0.001) amount of protein compared to other legumes. The cooked legumes showed higher fiber content. Cowpea and chickpea are rich in fiber compared to dal and mung bean. From a dietary point of view, cooked legumes can be added as a good source of dietary fiber to the daily diet. Ash content decreased in the cooked legumes; this may be due to the loss of minerals during soaking and boiling in water for a long time. The values obtained in our study are relatively lower than the values reported in a related study [10] but quite similar to the values reported in some studies [14, 21].

### 3.4. Nonvegetables

This food category is considered muscle foods. The significant contribution of muscle foods to human nutrition and their impact on health have been examined from different studies. Cooking these foods before consumption is necessary to improve digestibility and hygienic and sensory quality by inactivating pathogenic microorganisms. The results of the proximate composition of raw and cooked muscle foods are given in Table 5. Changes in proximate compositions in raw and cooked muscle foods were found to be significant (*p* < 0.001). The cooking temperature and time are important factors that affect the characteristics of cooked food. In Sri Lanka, the most common cooking methods are boiling and poaching. Poaching involves cooking foods in a cooking medium (water or milk) at lower temperatures (70 to 80°C). The moisture content of raw nonvegetable foods varied from 77.28 g per 100 g of DW in fish (large) to 2.20 g per 100 g of DW in sprats. Cooking has increased the moisture levels in some foods (sprats) while decreasing in some foods (dry fish, chicken, fish, egg, and salmon). All samples had a meager amount of carbohydrates, and considerable ash content was observed in this food category.

Among all nonvegetables, protein, fat, and ash contents were highest in the dry fish. Nonvegetables were found to be rich in proteins. Large fish (raw) had the lowest protein content (11.25 g per 100 g of DW), whereas dry fish (raw) had the highest protein content (31.62 g per 100 g of DW). However, cooking has reduced the protein content in most foods. Additionally, conventional cooking with heat denatures the tissue protein and makes it easier to digest. During cooking, the loss of protein is expected to occur in muscle foods [30]. Also, these foods are rich in fats. As in the other food categories, cooking significantly increased the fat content of the nonvegetables. Sprats had the lowest fat content (4.20 g per 100 g of DW) of all. The addition of nonvegetarian food to the daily diet, at least one unit, will help to get the proper nutrition needed for body growth. Fish is considered a better source of protein and healthy lipids. Fish provides the long-chain polyunsaturated omega-3-fatty acid, which might favorably improve lipid profile and reduce cholesterol levels and the risk of coronary heart diseases [22].

### 3.5. Cereal-Based Foods and Others

Foods analyzed under this category can be identified as typical breakfast meals widely consumed by locals. The results of the proximate composition of this food category are given in Table 6. Bread is a rich source of carbohydrates. Roti has the highest content of fat compared to bread and noodles. The incorporation of coconut scrapings in preparing the roti may have a significant impact on its fat content. Compared to the wheat bread and roti prepared using wheat flour, rice noodles would be a healthier option for regular consumption with a lower energy intake. However, preparing these foods with kurakkan flour may further improve their nutrition profile by adding more dietary fiber and lowering the carbohydrate content [12].

**Figure 3:**
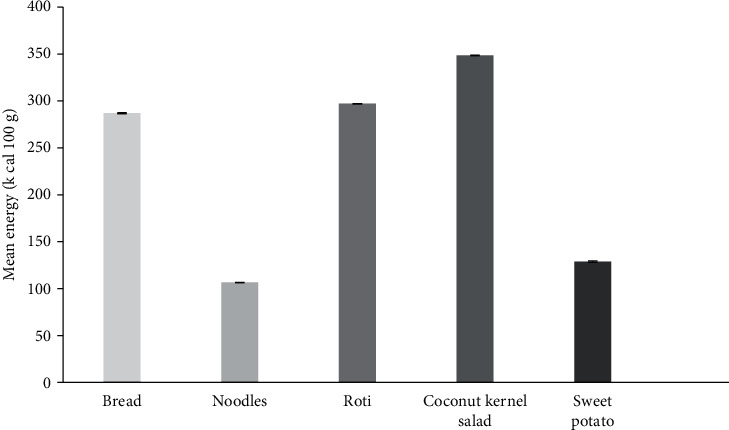
Energy values of the selected commonly consumed cereal-based food items and others. Results represent the mean ± SD (*n* = 6) at a 5% level of significance.

As illustrated in Figure 3, other commonly consumed category food items would fulfill the daily required energy. Coconut kernel sambol is a traditional Sri Lankan food, and it is frequently used as an accompaniment with rice, bread, and string hoppers by locals in their daily diet. As a whole, it consists of good content of protein and fiber. From the nutritional point of view, it can be identified as a good source of energy.

### 3.6. The Overall Effect of Cooking

The macro- and micronutrient content in food varies widely. Most of the time, foods are processed using different methods before consumption. In Sri Lanka, milk curries and tempered foods are common. It is evident from the results that there is a significant increase in the fat content in cooked food due to the use of CM, CO, or coconut scraps. It may be probably due to the temperature applied during the cooking process which facilitates the interaction between the food and the cooking medium, which can cause fats to penetrate the food. Vegetables are cooked with coconut milk until they achieved a soft consistency and palatability. Nevertheless, most of the time, other cereals and legumes are cooked by boiling and directly consumed accompanied with coconut scraps. However, the effect of Sri Lankan common culinary practices on the nutritional composition is poorly documented.

Coconut fats account for 80% of the fat intake among Sri Lankans [5]. Hence, in planning a healthy diet, the fat content incorporated into food due to the addition of coconut-based ingredients has to be taken into consideration. This is the first study to report on the effect of cooking with CM, CO, and scraped coconut on the nutritional status of commonly consumed food. Cooking incorporates desired characteristics to every food while a series of chemical and physical changes take place. These changes differ depending on the type of food being cooked and the cooking method. Due to these changes, some characteristics of the foods may improve, including flavor, texture, odor, and color. CO and CM can be added to foods to increase the energy density of the daily diet.

## 4. Conclusion

Vegetables and other types of food intake in diverse combinations are necessary for maintaining a healthy life and normal body functions. The changes in the nutritional value of vegetables and other foods due to cooking were evident from the changes in the macronutrient composition. The proximate composition of cooked food serves as a realistic parameter in assessing the nutritional status of food consumed. The present study serves as a guide in formulating health-related diets and in recommending the appropriate methods of cooking.

## Figures and Tables

**Table 1:** Vegetables and green leaves collected for the study, their common name, and edible plant part.

Botanical name	Vernacular names (Sinhalese and English)	Plant part used
*Cucurbita maxima*	Wattakka/pumpkin	Fruit
*Momordica charantia*	Karawila/bitter gourd	Fruit
*Beta vulgaris ssp*	Rathu ala/beetroot	Root
*Allium porrum*	Leek	Leaves
*Phaseolus vulgaris*	Bonchi/green bean	Fruit
*Brassica oleracea* var. *gongylodes*	Knolkhol/turnip	Root
*Brassica oleracea* var. *capitata*	Gowa/cabbage	Leaves
*Daucus carota*	Carrot	Root
*Brassica oleracea var. capitata*	Mal gowa/cauliflower	Flower
*Moringa oleifera*	Murunga/drumstick	Fruit
*Solanum melongena var. esculentum*	Wambatu/eggplant	Fruit
*Luffa acutangula*	Wetakolu/lufa	Fruit
*Nelumbo nucifera*	Nelum ala/lotus root	Root
*Abelmoschus esculentus*	Bandakka/okra	Fruit
*Capsicum annuum*	Malu miris/banana pepper	Fruit
*Solanum tuberosum*	Ala/potato	Root
*Raphanus sativus*	Rabu/radish	Root
*Phaseolus vulgaris sp.*	Maa karal/string bean	Fruit
*Trichosanthes cucumerina*	Pathola/snake gourd	Fruit
*Musa* spp.	Alu kesel/ash plantain	Fruit
*Ipomoea batatas*	Bathala/sweet potato	Root
*Musa acuminate Colla*	Kesel muwa/banana blossom	Flower
*Lasia spinosa*	Kohila/lassia	Stem
*Centella asiatica*	Gotukola/centrella	Leaves
*Sesbania grandiflora*	Kathurumurunga/hummingbird	Leaves
*Alternanthera sessilis*	Mukunuwenna/amaranthus	Leaves
*Spinacia oleracea*	Niwithi/spinach	Leaves

**Table 2:** Result of the proximate analysis of the selected vegetables (g/100 g of DW).

Food	Moisture (g)	Protein (g)	Fat (g)	Ash (g)	Fiber (g)	Carbohydrate (g)
*Pumpkin*						
Raw	92.6 ± 0.00	1.24 ± 0.01	0.10 ± 0.00	0.86 ± 0.00	0.79 ± 0.01	4.43 ± 0.02
Cooked (milk curry)	87.76 ± 0.06	0.82 ± 0.03	4.20 ± 0.03	0.49 ± 0.06	1.13 ± 0.23	5.58 ± 0.28
*p* value	0.001	0.001	0.001	0.008	0.064^∗^	0.002
*Bitter gourd*						
Raw	93.40 ± 0.01	1.24 ± 0.01	0.18 ± 0.00	0.86 ± 0.00	1.85 ± 0.06	2.29 ± 0.06
Cooked (tempered)	86.60 ± 0.01	0.87 ± 0.02	8.56 ± 0.05	0.80 ± 0.01	2.41 ± 0.03	0.76 ± 0.02
*p* value	0.001	0.001	0.001	0.001	0.001	0.001
*Beetroot*						
Raw	90.95 ± 0.01	1.61 ± 0.01	0.16 ± 0.00	1.13 ± 0.06	2.54 ± 0.31	3.63 ± 0.33
Cooked (milk curry)	88.40 ± 0.00	1.68 ± 0.06	3.93 ± 0.20	0.99 ± 0.01	2.91 ± 0.02	2.09 ± 0.10
*p* value	0.001	0.081^∗^	0.001	0.012	0.103^∗^	0.002
*Leek*						
Raw	90.78 ± 0.01	1.57 ± 0.01	0.33 ± 0.20	1.14 ± 0.01	1.80 ± 0.00	4.37 ± 0.01
Cooked (tempered)	85.60 ± 0.00	1.22 ± 0.05	4.32 ± 0.34	0.42 ± 0.04	2.86 ± 0.23	3.33 ± 0.18
*p* value	0.001	0.001	0.001	0.001	0.001	0.001
*Green bean*						
Raw	90.68 ± 0.01	1.83 ± 0.02	0.21 ± 0.01	0.66 ± 0.02	2.70 ± 0.11	3.92 ± 0.13
Cooked (milk curry)	87.10 ± 0.01	1.44 ± 0.04	4.32 ± 0.03	0.42 ± 0.01	2.87 ± 0.23	3.33 ± 0.18
*p* value	0.001	0.001	0.001	0.001	0.331^∗^	0.01
*Knolkhol*						
Raw	90.17 ± 0.06	1.80 ± 0.01	0.10 ± 0.00	1.20 ± 0.00	3.72 ± 0.30	3.02 ± 0.30
Cooked (milk curry)	88.70 ± 0.00	1.71 ± 0.05	3.09 ± 0.02	1.19 ± 0.06	4.33 ± 0.25	0.87 ± 0.36
*p* value	0.001	0.062^∗^	0.001	0.851^∗^	0.053^∗^	0.001
*Cabbage*						
Raw	90.44 ± 0.00	1.24 ± 0.01	0.91 ± 0.02	0.70 ± 0.01	2.23 ± 0.06	4.47 ± 0.07
Cooked (tempered)	81.27 ± 0.01	1.05 ± 0.04	9.11 ± 0.04	0.53 ± 0.05	2.97 ± 0.12	5.07 ± 0.19
*p* value	0.001	0.001	0.001	0.003	0.001	0.006
*Carrot*						
Raw	88.39 ± 0.01	1.06 ± 0.01	0.22 ± 0.01	0.92 ± 0.02	2.87 ± 0.23	6.54 ± 0.24
Cooked (milk curry)	90.94 ± 0.00	0.68 ± 0.02	3.82 ± 0.03	0.66 ± 0.02	3.62 ± 0.03	0.28 ± 0.04
*p* value	0.001	0.001	0.001	0.001	0.005	0.001
*Cauliflower*						
Raw	89.19 ± 0.01	3.01 ± 0.01	0.31 ± 0.01	0.83 ± 0.03	3.07 ± 0.12	3.60 ± 0.16
Cooked (curry)	82.59 ± 0.01	1.98 ± 0.04	9.20 ± 0.04	0.62 ± 0.02	3.63 ± 0.06	1.97 ± 0.06
*p* value	0.001	0.001	0.001	0.001	0.002	0.001
*Drumstick*						
Raw	87.79 ± 0.01	2.22 ± 0.02	0.21 ± 0.01	0.98 ± 0.03	3.30 ± 0.26	5.50 ± 0.24
Cooked (milk curry)	88.20 ± 0.00	1.98 ± 0.04	3.13 ± 0.02	1.10 ± 0.01	4.53 ± 0.06	1.04 ± 0.09
*p* value	0.001	0.001	0.001	0.003	0.001	0.001
*Eggplant*						
Raw	92.60 ± 0.00	0.97 ± 0.01	0.59 ± 0.01	0.59 ± 0.01	3.00 ± 0.20	2.25 ± 0.22
Cooked (tempered)	78.18 ± 0.01	0.87 ± 0.06	11.49 ± 0.03	1.01 ± 0.01	3.57 ± 0.06	4.78 ± 0.08
*p* value	0.001	0.348^∗^	0.001	0.001	0.009	0.001
*Lufa*						
Raw	93.79 ± 0.01	1.37 ± 0.02	0.21 ± 0.01	0.41 ± 0.01	1.38 ± 0.08	2.84 ± 0.08
Cooked (milk curry)	87.80 ± 0.00	1.00 ± 0.06	4.34 ± 0.04	0.86 ± 0.01	2.73 ± 0.23	3.26 ± 0.15
*p* value	0.001	0.001	0.001	0.001	0.001	0.012
*Lotus root*						
Raw	79.40 ± 0.00	2.57 ± 0.02	0.09 ± 0.01	0.93 ± 0.03	4.83 ± 0.21	12.18 ± 0.25
Cooked (curry)	81.79 ± 0.01	1.64 ± 0.07	8.04 ± 0.06	0.91 ± 0.01	6.43 ± 0.06	1.19 ± 0.09
*p* value	0.001	0.001	0.001	0.346^∗^	0.003	0.001
*Mushroom*						
Raw	91.80 ± 0.01	3.24 ± 0.00	0.30 ± 0.01	0.80 ± 0.02	1.00 ± 0.00	2.83 ± 0.07
Cooked (curry)	72.20 ± 0.01	2.27 ± 0.09	11.56 ± 0.01	1.01 ± 0.04	2.10 ± 0.10	10.58 ± 0.12
*p* value	0.001	0.001	0.001	0.001	1	0.001
*Okra*						
Raw	89.72 ± 0.02	1.87 ± 0.01	0.16 ± 0.00	0.90 ± 0.00	3.20 ± 0.00	4.15 ± 0.01
Cooked (tempered)	82.58 ± 0.02	1.93 ± 0.05	5.13 ± 0.03	0.86 ± 0.01	3.97 ± 0.06	5.54 ± 0.12
*p* value	0.001	0.089^∗^	0.001	0.001	0.001	0.001
*Banana pepper*						
Raw	91.80 ± 0.01	1.95 ± 0.02	0.70 ± 0.01	0.80 ± 0.00	3.45 ± 0.09	1.29 ± 0.11
Cooked (tempered)	84.00 ± 0.01	0.92 ± 0.08	9.83 ± 0.03	0.84 ± 0.03	3.97 ± 0.06	0.44 ± 0.07
*p* value	0.001	0.001	0.001	0.06	0.001	0.001
*Potato*						
Raw	68.70 ± 0.00	2.14 ± 0.01	0.20 ± 0.01	1.07 ± 0.06	2.07 ± 0.11	25.82 ± 0.15
Cooked (milk curry)	76.44 ± 0.03	1.71 ± 0.08	6.15 ± 0.01	0.92 ± 0.06	2.54 ± 0.05	12.24 ± 0.22
*p* value	0.001	0.001	0.001	0.037	0.003	0.001
*Radish*						
Raw	95.79 ± 0.01	1.04 ± 0.01	0.08 ± 0.00	0.91 ± 0.02	1.27 ± 0.12	0.90 ± 0.11
Cooked (tempered)	81.40 ± 0.00	0.56 ± 0.13	10.93 ± 0.03	0.99 ± 0.01	1.72 ± 0.08	4.39 ± 0.18
*p* value	0.001	0.003	0.001	0.004	0.005	0.001
*String bean*						
Raw	90.58 ± 0.03	2.10 ± 0.01	0.30 ± 0.01	1.74 ± 0.01	5.03 ± 0.06	0.25 ± 0.07
Cooked (tempered)	74.50 ± 0.02	2.70 ± 0.10	12.07 ± 0.06	1.28 ± 0.01	5.80 ± 0.00	3.65 ± 0.16
*p* value	0.001	0.001	0.001	0.001	0.001	0.001
*Snake gourd*						
Raw	88.78 ± 0.01	1.78 ± 0.01	0.10 ± 0.01	0.71 ± 0.01	3.60 ± 0.00	5.03 ± 0.01
Cooked (milk curry)	83.80 ± 0.01	1.88 ± 0.16	5.30 ± 0.01	0.59 ± 0.01	3.95 ± 0.05	4.46 ± 0.21
*p* value	0.001	0.328^∗^	0.001	0.001	0.001	0.01
*Ash plantain*						
Raw	90.80 ± 0.00	0.56 ± 0.01	0.10 ± 0.00	0.96 ± 0.01	3.23 ± 0.21	5.35 ± 1.60
Cooked (milk curry)	91.39 ± 0.35	0.43 ± 0.01	3.01 ± 0.02	1.15 ± 0.05	3.77 ± 0.06	0.45 ± 0.08
*p* value	0.043	0.01	0.001	0.003	0.013	0.006
*Banana blossom*						
Raw	90.20 ± 0.02	1.98 ± 0.01	0.10 ± 0.00	0.71 ± 0.00	6.13 ± 0.12	0.88 ± 0.12
Cooked (tempered)	78.60 ± 0.03	1.92 ± 0.02	9.42 ± 0.03	1.38 ± 0.03	7.50 ± 0.10	1.17 ± 0.08
*p* value	0.001	0.009	0.001	0.001	0.001	0.024
*Lassia*						
Raw	74.70 ± 0.04	1.77 ± 0.00	0.10 ± 0.00	0.81 ± 0.01	22.07 ± 0.06	0.52 ± 0.01
Cooked (curry)	63.70 ± 0.02	1.85 ± 0.01	4.51 ± 0.17	0.67 ± 0.01	28.00 ± 0.10	1.35 ± 0.13
*p* value	0.001	0.001	0.001	0.001	0.001	0.001

**Table 3:** Result of the proximate analysis of the selected green leafy vegetables (g/100 g of DW).

Food	Moisture (g)	Protein (g)	Fat (g)	Ash (g)	Fiber (g)	Carbohydrate (g)
*Centella*						
Raw	81.4 ± 0.00	1.81 ± 0.02	4.21 ± 0.00	3.74 ± 0.00	8.15 ± 0.00	0.69 ± 0.02
Cooked	68.50 ± 0.01	1.34 ± 0.03	5.92 ± 0.07	6.81 ± 0.01	16.57 ± 0.06	0.86 ± 0.03
*p* value	0.001	0.001	0.001	0.001	0.001	0.001
*Sesbania*						
Raw	80.40 ± 0.01	1.97 ± 0.00	4.63 ± 0.03	3.15 ± 0.00	9.13 ± 0.00	0.72 ± 0.03
Cooked	69.10 ± 0.03	2.15 ± 0.01	5.45 ± 0.05	7.42 ± 0.02	14.82 ± 0.03	1.06 ± 0.08
*p* value	0.003	0.001	0.001	0.001	0.001	0.002
*Spinach*						
Raw	67.78 ± 0.01	1.54 ± 0.01	9.44 ± 0.02	4.34 ± 0.01	14.23 ± 0.06	2.66 ± 0.05
Cooked	58.60 ± 0.01	1.68 ± 0.02	10.33 ± 0.01	6.34 ± 0.00	15.62 ± 0.03	7.44 ± 0.06
*p* value	0.001	0.001	0.001	0.001	0.001	0.001
*Amaranthus*						
Raw	81.80 ± 0.01	2.08 ± 0.01	2.79 ± 0.02	2.66 ± 0.01	10.07 ± 0.00	0.61 ± 0.03
Cooked	75.70 ± 0.02	1.96 ± 0.01	3.67 ± 0.06	3.49 ± 0.13	14.21 ± 0.01	1.04 ± 0.05
*p* value	0.001	0.001	0.001	0.001	0.001	0.001

Data are expressed as the mean ± SD. Values are an average of at least three replicate experiments and calculated on a dry weight basis for all the constituents except for moisture. Values before and after cooking of each food show a significant difference (*p* < 0.001) at a 5% level of significance.

**Table 4:** Result of the proximate analysis of cereals and legumes (g/100 g of DW).

Food	Moisture (g)	Protein (g)	Fat (g)	Ash (g)	Fiber (g)	Carbohydrate (g)
*Dal*						
Raw	51.70 ± 0.00	21.56 ± 0.48	1.01 ± 0.01	3.34 ± 0.01	1.24 ± 0.04	20.87 ± 0.03
Cooked (milk curry)	57.39 ± 0.01	15.81 ± 0.01	1.72 ± 0.01	2.68 ± 0.03	1.97 ± 0.21	20.45 ± 0.20
*p* value	0.001	0.001	0.001	0.001	0.004	0.023
*Mung bean*						
Raw	89.66 ± 0.00	3.16 ± 0.01	0.16 ± 0.01	0.46 ± 0.01	1.72 ± 0.08	4.85 ± 0.08
Cooked (boiled)	93.19 ± 0.01	2.32 ± 0.00	0.12 ± 0.02	0.92 ± 0.02	2.06 ± 0.05	1.39 ± 0.06
*p* value	0.001	0.001	0.008	0.001	0.003	0.001
*Cowpea*						
Raw	12.58 ± 0.00	22.19 ± 0.00	1.16 ± 0.01	3.21 ± 0.01	8.60 ± 0.03	52.26 ± 0.04
Cooked (boiled)	68.18 ± 0.03	7.93 ± 0.01	0.58 ± 0.02	0.95 ± 0.01	8.85 ± 0.06	13.52 ± 0.04
*p* value	0.001	0.001	0.001	0.001	0.001	0.001
*Chickpea*						
Raw	12.60 ± 0.01	19.26 ± 0.01	6.27 ± 0.11	2.86 ± 0.01	6.50 ± 0.01	42.41 ± 0.13
Cooked (boiled)	60.60 ± 0.02	7.35 ± 0.00	2.61 ± 0.01	0.94 ± 0.01	7.41 ± 0.02	21.09 ± 0.03
*p* value	0.001	0.001	0.001	0.001	0.001	0.001

Data are expressed as the mean ± SD. Values are an average of at least three replicate experiments and calculated on a dry weight basis for all the constituents except for moisture. Values before and after cooking of each food show a significant difference (*p* < 0.001) at a 5% level of significance.

**Table 5:** Result of the proximate analysis of nonvegetarian foods (g/100 g of DW).

Food	Moisture (g)	Protein (g)	Fat (g)	Ash (g)	Fiber (g)	Carbohydrate (g)
*Dry fish*						
Raw	10.60 ± 0.00	31.12 ± 0.01	12.21 ± 0.01	3.47 ± 0.02	ND	42.60 ± 0.03
Cooked (tempered)	7.79 ± 0.01	28.90 ± 0.02	16.30 ± 0.03	3.34 ± 0.01	ND	43.67 ± 0.02
*p* value	0.001	0.001	0.001	0.001	—	0.001
*Sprats*						
Raw	2.20 ± 0.00	16.41 ± 0.00	4.20 ± 0.00	4.82 ± 0.00	ND	72.37 ± 0.00
Cooked (tempered)	12.60 ± 0.00	15.32 ± 0.00	5.64 ± 0.03	5.34 ± 0.03	ND	61.01 ± 0.06
*p* value	—	—	0.001	0.001	—	0.001
*Chicken*						
Raw	66.52 ± 0.00	12.32 ± 0.00	8.63 ± 0.06	4.07 ± 0.06	2.51 ± 0.02	5.94 ± 0.13
Cooked (curry)	45.57 ± 0.02	7.38 ± 0.03	9.64 ± 0.03	4.31 ± 0.03	2.81 ± 0.01	29.32 ± 0.04
*p* value	0.001	0.001	0.001	0.005	0.001	0.001
*Fish (large)*						
Raw	77.28 ± 0.00	11.25 ± 0.00	8.86 ± 0.00	2.53 ± 0.02	ND	0.07 ± 0.00
Cooked (curry)	58.54 ± 0.07	9.12 ± 0.02	9.47 ± 0.05	2.72 ± 0.00	ND	20.18 ± 0.14
*p* value	0.001	0.001	0.001	0.001	—	0.001
*Fish (small)*						
Raw	75.41 ± 0.00	12.31 ± 0.01	9.41 ± 0.02	2.82 ± 0.01	ND	0.06 ± 0.01
Cooked (curry)	59.35 ± 0.05	11.60 ± 0.00	9.83 ± 0.02	3.11 ± 0.01	ND	16.11 ± 0.06
*p* value	0.001	0.001	0.001	0.001	—	0.001
*Egg*						
Raw	76.60 ± 0.00	12.27 ± 0.01	9.45 ± 0.02	1.01 ± 0.01	ND	0.68 ± 0.04
Cooked (boiled)	74.59 ± 0.01	12.48 ± 0.02	10.34 ± 0.01	1.04 ± 0.03	ND	1.55 ± 0.02
*p* value	0.001	0.001	0.001	0.242^∗^	—	0.001
*Salmon*						
Raw	65.87 ± 0.01	21.93 ± 0.00	9.91 ± 0.01	2.28 ± 0.00	ND	0.01 ± 0.00
Cooked (tempered)	28.80 ± 0.00	21.34 ± 0.00	10.02 ± 0.03	2.45 ± 0.04	ND	37.39 ± 0.07
*p* value	0.001	—	0.005	0.002	—	0.001

Data are expressed as the mean ± SD. Values are an average of at least three replicate experiments and calculated on a dry weight basis for all the constituents except for moisture. *p* values with an asterisk mark within the column do not exhibit a significant difference between raw and cooked vegetables (*p* > 0.05) at a 5% level of significance.

**Table 6:** Result of the proximate analysis of commonly consumed cereal-based foods and others (g/100 g of DW).

Food	Moisture (g)	Protein (g)	Fat (g)	Ash (g)	Fiber (g)	Carbohydrate (g)	Energy (g)
Sweet potato (boiled)	61.40 ± 0.00	1.13 ± 0.01	0.30 ± 0.00	0.94 ± 0.01	2.60 ± 0.00	33.62 ± 0.02	128.01 ± 0.94
Bread	36.13 ± 0.12	5.66 ± 0.06	3.26 ± 0.07	1.24 ± 0.01	2.24 ± 0.03	58.37 ± 0.08	285.45 ± 0.74
Noodles	72.53 ± 0.06	1.54 ± 0.01	0.72 ± 0.03	0.16 ± 0.01	1.70 ± 0.01	23.35 ± 0.06	106.01 ± 0.23
Roti	26.58 ± 0.01	10.45 ± 0.02	6.86 ± 0.03	2.26 ± 0.01	5.81 ± 0.01	48.05 ± 0.03	295.71 ± 0.15
Coconut sambol	45.80 ± 0.01	3.40 ± 0.01	32.71 ± 0.05	4.83 ± 0.04	3.54 ± 0.06	9.72 ± 0.06	346.89 ± 0.18

Data are expressed as the mean ± SD. Values are an average of at least three replicate experiments and calculated on a dry weight basis for all the constituents except for moisture. Values before and after cooking each food show a significant difference (*p* < 0.001) at a 5% level of significance.

## Data Availability

The data sets analyzed during the current study are available from the corresponding author on reasonable request (corresponding author email: bimalimadu123@gmail.com).
